# Inclusion of Safety-Related Issues in Economic Evaluations for Seasonal Influenza Vaccines: A Systematic Review

**DOI:** 10.3390/vaccines9020111

**Published:** 2021-02-02

**Authors:** Tanja Fens, Pieter T. de Boer, Eugène P. van Puijenbroek, Maarten J. Postma

**Affiliations:** 1 Department of PharmacoTherapy, Epidemiology & -Economics (PTE2), Groningen Research Institute of Pharmacy, University of Groningen, 9713 AV Groningen, The Netherlands; ptdeboer@hotmail.com (P.T.d.B.); E.P.van.Puijenbroek@rug.nl (E.P.v.P.); M.J.Postma@rug.nl (M.J.P.); 2 Department of Health Sciences, University Medical Center Groningen, University of Groningen, 9713 GZ Groningen, The Netherlands; 3 Netherlands Pharmacovigilance Centre Lareb, 5237 MH ’s-Hertogenbosch, The Netherlands; 4 Department of Economics, Econometrics & Finance, Faculty of Economics & Business, University of Groningen, 9747 AE Groningen, The Netherlands; 5 Department of Pharmacology and Therapy, Faculty of Medicine, Universitas Airlangga, Surabaya 60132, Indonesia; 6 Center of Excellence in Higher Education for Pharmaceutical Care Innovation, Universitas Padjadjaran, Bandung 45363, Indonesia

**Keywords:** seasonal influenza vaccines, economic evaluations, adverse events following immunization

## Abstract

(1) Background: Vaccines for seasonal influenza are a good preventive and cost-effective strategy. However, it is unknown *if* and *how* these economic evaluations include the adverse events following immunization (AEFI), and what the impact of such inclusion is on the health economic outcomes. (2) Methods: We searched the literature, up to January 2020, to identify economic evaluations of seasonal influenza vaccines that considered AEFIs. The review protocol was published in PROSPERO (CDR42017058523). (3) Results: A total of 52 economic evaluations considered AEFI-related parameters in their analyses, reflecting 16% of the economic evaluations on seasonal influenza vaccines in the initial study selection. Most studies used the societal perspective (64%) and evaluated vaccination of children (37%). Where considered, studies included direct medical costs of AEFIs (90%), indirect costs (27%), and disutilities/quality-adjusted life years loss due to AEFIs (37%). The majority of these studies accounted for the effects of the costs of AEFI on cost-effectiveness for Guillain–Barré syndrome. In those papers allowing cost share estimation, direct medical cost of AFEIs was less than 2% of total direct costs. (4) Conclusions: Although the overall impact of AEFIs on the cost-effectiveness outcomes was found to be low, we urge their inclusion in economic evaluations of seasonal influenza vaccines to reflect comprehensive reports for the decision makers and end-users of the vaccination strategies.

## 1. Introduction

Seasonal influenza spreads very easily among people from all age groups almost every year [1]. Although influenza is mostly self-limiting, serious complications can arise in vulnerable patient groups. Populations with higher risk of complications include pregnant women, patients with chronic medical conditions, children aged 6–60 months, and the elderly [1,2]. Vaccination is considered as the most effective way to prevent disease and/or severe outcomes from the illness [1]. Since 1945, influenza vaccines have been marketed and used as an efficient and cost-effective preventive tool [3].

There are several types of seasonal influenza vaccines available worldwide. Most common are inactivated influenza vaccines (IIVs), available as trivalent influenza vaccines (TIVs) or quadrivalent influenza vaccines (QIVs), and live attenuated influenza vaccines (LAIVs), which are nowadays only available as quadrivalent vaccines (Q-LAIVs). While these vaccines are produced with egg-based technology, there are also cell-based and recombinant influenza vaccines. More recent additions to the portfolio of vaccines concern the modification to high-dose and adjuvanted vaccines. All of these vaccines are approved for seasonal influenza and may be indicated for different age groups. Regarding the route of administration, most of the vaccines for preventing seasonal influenza are given intramuscularly, but also those to be applied intradermally, subcutaneously, or nasally exist [4,5]. The seasonal influenza vaccine is generally considered safe, yet it can sometimes cause adverse events following immunization (AEFIs). Generally, AEFIs are considered to be less serious as compared to influenza itself. In rare occasions, the vaccines have been shown to cause serious complications, such as Guillain–Barré syndrome (GBS) or severe allergic reactions such as anaphylactic reactions [6]. For example, AEFIs with IIV may include local reactions, such as pain, erythema, swelling, and/or systemic reactions, such as fever, headache, malaise, myalgia, fatigue, febrile seizures, syncope, anaphylaxis, paresthesia, and GBS [6,7]. Moreover, LAIV is associated with the following AEFI: runny nose or nasal congestion in all ages, fever, wheezing, headache, sore throat, tiredness/weakness, myalgia, cough, chills, and sinusitis, but also more serious such as GBS [6,7,8]. 

Guidelines for economic evaluations in healthcare, for example, in the Netherlands [9], Sweden [10], and Portugal [11], advise inclusion of all relevant costs and effects, irrespective of who faces and bears the costs, including those for AEFIs. Moreover, the recently published WHO guide on the economic evaluation of influenza vaccination suggests taking AEFIs into account when possible [12]. The National Institute for Health and Care Excellence (NICE) in the UK requires inclusion of the effects of adverse events on health-related quality of life, as well as costs for each adverse event in the process of any drug submission, inclusive vaccines [13]. It has been suggested that economic evaluations on seasonal influenza vaccine lack information on AEFI-related costs and effects [14]. While some studies potentially do include such costs and effects, non-consistent reporting hampers the comparison between studies. Furthermore, it may result in inadequate transparency and credibility for the decision makers and potential bias in results.

In this systematic review, we aim to assess if and how economic evaluations on seasonal influenza vaccines include AEFIs, and what would be the impact of its inclusion on the health economic outcomes. The outcomes of this review may contribute to future consistent and credible inclusion and reporting of AEFIs’ costs and effects in comprehensive economic evaluations of seasonal influenza vaccines, as indicated by the guidelines for economic evaluations and fully in line with the aim of economic evaluations to include benefits as well as harms of any pharmaceutical considered.

## 2. Materials and Methods

Our study followed the Preferred Reporting Items for Systematic Reviews and Meta-Analyses (PRISMA) guidelines [15], and accounting for the extended “harm” items applicable for systematic reviews only [16]. We developed a study protocol that was submitted to PROSPERO (CDR42017058523) [17]. We conducted our search in the scientific databases as listed below and performed additional search in the gray literature for completing all up-to-date information.

### 2.1. Eligibility Criteria

We considered only full economic evaluations, i.e., cost-effectiveness, cost–benefit, cost-minimization, or cost–utility studies, discussing seasonal influenza vaccines [18]. No limitations on age, gender, health condition, or population for inclusion were applied. Selected economic evaluations should have compared parameters between vaccinated and non-vaccinated populations, or populations vaccinated with different types of vaccines for seasonal influenza, for example, TIV vs. QIV, or concerned different routes of administration. The following types of studies were excluded: economic evaluations discussing treatments for seasonal influenza (for example, antiviral medication), studies addressing pandemic influenza vaccines, cost analyses, cost-of-illness analyses, burden-of-illness studies, partial/non-comparative economic evaluations, posters, and conference abstracts.

### 2.2. Information Sources and Search Strategy

We searched for full-text published studies in peer-reviewed journals. Our focus was on full economic evaluations of seasonal influenza vaccines. The search strategy was performed in 3 steps. First, we performed an initial limited search (using the following keywords: influenza, vaccine, seasonal, and economics) of MEDLINE in PubMed, followed by analysis of the text words contained in the title, the abstract, and the index terms used to describe the articles. This was used to create a detailed search strategy given in Table 1, including 4 search lines, each composed of Medical Subject Headings (MeSH) terms, as well as other, previously identified index terms.

In the second step, we ran the 4 search lines (as presented in Table 1) in MEDLINE, EMBASE, and the Cochrane Library to search for articles of interest. In the third step, we searched the reference lists of all identified reports and articles for additional studies. We also checked gray literature (Google search, governmental and research reports), and searched additional databases indexing economic evaluations, such as National Institute for Health Research Economic Evaluation Database (NHS EED) and Health Economic Evaluations Database and Cost Effectiveness Analysis registry (HEED CEA), to ensure a comprehensive coverage. Within these databases, applying the detailed search strategy was not possible, and therefore we performed searches using only the key words “influenza vaccines” and assessed those results for adherence to our selection criteria in order to add eligible articles for a final review. Studies published until January 2020 were considered for inclusion in this review. No language limitation was applied.

### 2.3. Study Selection and Data Collection Process

Two researchers, T.F. and P.T.d.B., independently assessed the search results—1152 articles. Disagreements were discussed and consensus was reached with the help of M.J.P.

#### 2.3.1. Study Selection

The search results were examined in 2 rounds, using a unified study selection form (Appendix A, Table A1) that included 5 selection criteria questions. In the first round of title/abstract screening, 4 out of the 5 selection questions were considered to identify the eligible economic evaluations. In the second round of full text screening, we addressed the fifth question as well, which allowed us to identify the economic evaluations that discuss AEFIs. Agreement/reliability was calculated using kappa statistics [19].

#### 2.3.2. Data Extraction and Data Items

Data extraction was conducted using a pre-designed data extraction form (Supplementary Materials, Table S1) by the review team. This form includes information about study identification, type of economic evaluation, study perspective, type of vaccine used and its comparator, population characteristic (e.g., health condition, gender and age), location/geographical information, settings, time period (year/time frame), cost data (total and AEFI-related costs), currency used, pricing period/date, AEFIs considered, other AEFI-related data (e.g., health related quality of life due to AEFI, frequency of AEFI-occurrence and duration of AEFI), data sources, funding, authors’ conclusions, and other relevant data identified as significant for inclusion during the article screening.

#### 2.3.3. Outcomes

The primary outcomes considered AEFI-related costs and health effects, for example, the impact on utilities or health-related quality of life. Cost outcomes were expressed in 2020 price year levels using the Campbell and Cochrane Economics Methods Group and the Evidence for Policy and Practice Information and Coordination Centre web-based tool [20] to enhance comparability between studies. The percentage shares of AEFI-related costs in the total costs presented in the economic evaluations were studied as a secondary outcome.

According to the WHO, the AEFI is any untoward medical occurrence that follows immunization and that does not necessarily have a causal relationship with the usage of the vaccine [21]. The term AEFI was chosen to be used for this study in order to avoid misinterpretations due to the diverse presentation of the safety-related parameters within the studies, as investigating the casualty was not within the aim of this review.

### 2.4. Risk of Bias/Quality Assessment

After the study selection, 2 researchers, T.F. and P.T.d.B., assessed the quality of the study and the risk of bias. For the methodological quality, we used the Consensus Health Economic Criteria (CHEC), an extended list for conducting systematic reviews on the basis of economic evaluation studies [22,23]. This 20-question structure from the CHEC-extended list was filled with agreements and disagreements, as the list contains only yes/no questions. In case of insufficient information, or lack of clarity, we answered negatively, which does not indicate a complete exclusion of the concerned issue. When we had agreed that the issue in question was sufficiently described, we selected “yes”. Disagreements regarding this assessment were resolved by involving a third researcher (M.J.P.). For better presentation of the outcomes, we inputted the questions from this checklist in the review production tool RevMan 5.3 (Review Manager 5.3, The Cochrane Collaboration, London, United Kingdom) [24]. This tool allows for selecting low/high/unclear risk of bias. Considering our checklist, we used “low risk” for our positive answers, “high risk” of bias for our negative answers, and “unclear risk” for items not applicable for the evaluated study.

## 3. Results

### 3.1. Study Selection

**Figure 1:**
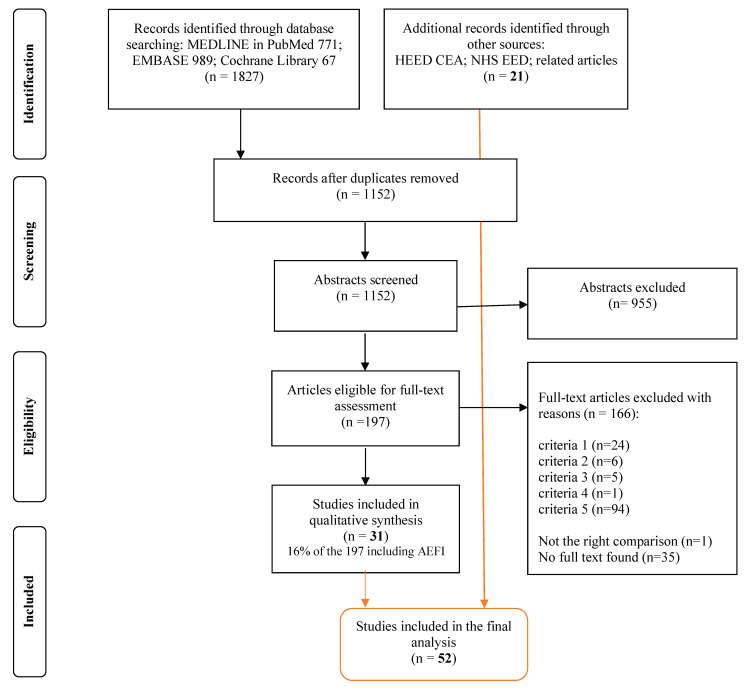
Preferred Reporting Items for Systematic Reviews and Meta-Analyses (PRISMA) flow diagram. NHS EED—National Institute for Health Research Economic Evaluation Database; HEED CEA—Health Economic Evaluations Database and Cost-Effectiveness Analysis registry; CEA—cost-effectiveness analysis; CUA—cost–utility analysis; CMA—cost-minimization analysis; CBA—cost–benefit analysis; EE—economic evaluation; AEFI—adverse event following immunization. This diagram shows the study selection process in steps, starting from database search, then removing duplicates, selecting by abstract screening, and full-text screening. The listed criteria for eliminating the full-text articles concern the following questions: (1) Is the article a full economic evaluation study (designs to be considered: CMA, CEA, CBA, or CUA)? (2) Is the intervention a vaccination? (3) Is the vaccine used for seasonal influenza? (4) Are the outcome measures economic parameters? (5) Does this EE discuses AEFI? Bold: 31+21 = 52, the total number of reviewed studies.

We identified 1827 articles, from which 771 were in MEDLINE, 989 in EMBASE, and 67 in Cochrane Library. The duplicates were removed using RefWorks and further manual scanning of the abstracts, and this resulted in 675 duplicates from the three databases. The resulting number of unique articles for title/abstract selection was 1152. After the first round of title/abstract scanning (accounting for the first four questions of our study selection form given in Appendix A, Table A1), we identified 197 potentially suitable papers, which we further explored within their full text (with the fifth question of the study selection form given in Appendix A, Table A1) for AEFI inclusion. The kappa statistic calculations resulted in a coefficient of 0.98, indicating good reliability according to Landis and Koch [25] and very good according to Altman’s [26] interpretation (calculations are given in Appendix A, Table A2). This search, performed in the three above-mentioned databases, resulted in 31 articles eligible for our review (16% of the 197 full-text scanned papers). After searching the additional databases indexing economic evaluations as mentioned in the methods section, we added 21 articles that were eligible to be included in the review and adhering to the selection criteria. These studies were not presented through the steps of selection, as they were not identified using the detailed search strategy (given in Table 1), and therefore the 21 eligible studies from the additional search were directly added to the 31 eligible studies from the main database search. Finally, this review includes 52 (31 from the main search plus 21 from the additional search) full economic studies discussing safety/AEFI-related outcomes. The flow diagram of the study selection is presented in Figure 1.

### 3.2. Study Characteristics

The general study characteristics and the different ways our reviewed studies reflected these characteristics are summarized in Table 2, including the percentage of studies reflecting those specific characteristics in the total reviewed articles. Further details regarding the extracted data, as well as the AEFI definitions, AEFI sources, ways the AEFI were ascertained, and in what time period, can be found in Supplementary Materials, Table S1.

#### 3.2.1. Type of Economic Evaluation and Study Perspective

The majority of the evaluated studies reported their outcomes using CUA [27,28,29,30,31,32,33,34,35,36,37,38,39,40,41,42,43,44,45,46]. Other types of economic evaluation considered were CEAs (*N* = 11) [47,48,49,50,51,52,53,54,55,56,57], CBA (11 studies) [58,59,60,61,62,63,64,65,66,67,68], or combining more than one type (10 studies) [69,70,71,72,73,74,75,76,77,78]. Most studies reflected the societal perspective (*N* = 18) [30,34,35,36,41,42,43,45,46,47,55,57,63,64,67,73,78,79]. Fifteen studies evaluated the issue in question from more than one perspective, combining the societal perspective with another perspective [27,32,33,37,38,39,40,44,49,52,56,59,66,68,77]. The rest of the studies considered either a healthcare provider perspective [29,31,50,58,72,75], employer perspective [60,65], patient perspective [51], family perspective [69], school perspective [70], net monetary benefit perspective [71], or medical insurance perspective [61].

#### 3.2.2. Compared Vaccine Alternatives and Vaccinated Population

The reviewed articles were mostly considering comparison of either IIV (*N* = 44) or LAIV (*N* = 8) with no vaccination, or another vaccine alternative. More details on the compared alternatives per article are presented in Table 3.

Population characteristics showed that analyses focusing on children were most frequent (*N* = 18) [27,28,30,33,37,42,47,48,49,52,55,56,67,69,70,76,77,78], followed by analyses of the elderly (*N* = 12) [35,38,39,40,46,50,53,57,58,62,72,75], pregnant/postpartum women and infants (*N* = 7) [29,31,32,34,41,59,74] and other groups of adults (workforce, risk groups) [36,43,44,45,51,54,60,61,63,64,65,66,68,71,73].

#### 3.2.3. Study Location and Settings

The majority of the studies (*N* = 29) concerned the U.S. context [30,32,34,35,36,37,38,39,40,41,42,43,45,46,48,51,54,55,60,63,64,66,67,70,73,74,76,78]. Considering the study settings, we identified model-based studies (32) [27,28,29,30,31,32,33,34,36,37,38,39,40,41,42,45,46,47,48,49,50,51,58,59,60,63,66,67,69,73,74,75], and trial-based and observational/survey studies (20) [35,43,44,52,53,54,55,56,57,61,62,64,65,66,68,70,71,72,77,78].

#### 3.2.4. Data Sources and Funding

Data about the costs of AEFIs were derived from publications and public databases. For example, few studies [59,73,77,78] used cost data based on the database (The Medstat Group) reporting payments for health insurance companies in the USA [42,43]. The IBM Micromedex RED BOOK [80] was also often used as a cost reference source [30,32,36,37,38,39,40,42,49,51,55,78]. Health impact data were mostly taken from population-based studies and surveys (utilities/quality-adjusted life years (QALYs)) [53,81], and one study [39] used an estimate for the QALYs. Clinical trials were mostly the source for probabilities/frequencies. The majority of the funded studies were financed by national or international health institutions, while only 15% of those studies received financial support from the pharmaceutical industry.

### 3.3. AEFI-Related Outcomes

The reviewed articles accounted for the AEFI in a matter of direct and indirect costs, disutility, duration, and probability/frequency of occurrence. Table 2 displays the proportions of articles accounting for each of the previously mentioned issues. The inclusion of these parameters per article, as well as the AEFIs accounting for the costs are given in Table 3. Notably, from the severe AEFIs, the GBS was most often included (29% of the studies), both in evaluations of IIV and LAIV [29,34,36,41,42,43,47,54,59,63,67,73,74,76,78]. Six of the articles [43,59,67,73,76,78] accounting for GBS also reported anaphylaxis, mainly when vaccinating children [59,67,76,78]. Furthermore, medically significant wheezing (MSW) was reported only in children, vaccinated with LAIV [42,55,78]. For the local AEFI, over-the-counter treatments were used, such as paracetamol (acetaminophen), ketoprofen, fluocinolone to release mild-to-moderate pain [32,33,36,38,39,40,41,55,56,60,62,69,70,72,75,78], pseudoephedrine as nasal decongestant [27,78], or prescription medication such as the albuterol inhaler for management of wheezing in children [78].

#### 3.3.1. AEFI-Related Costs

**Figure A1:**
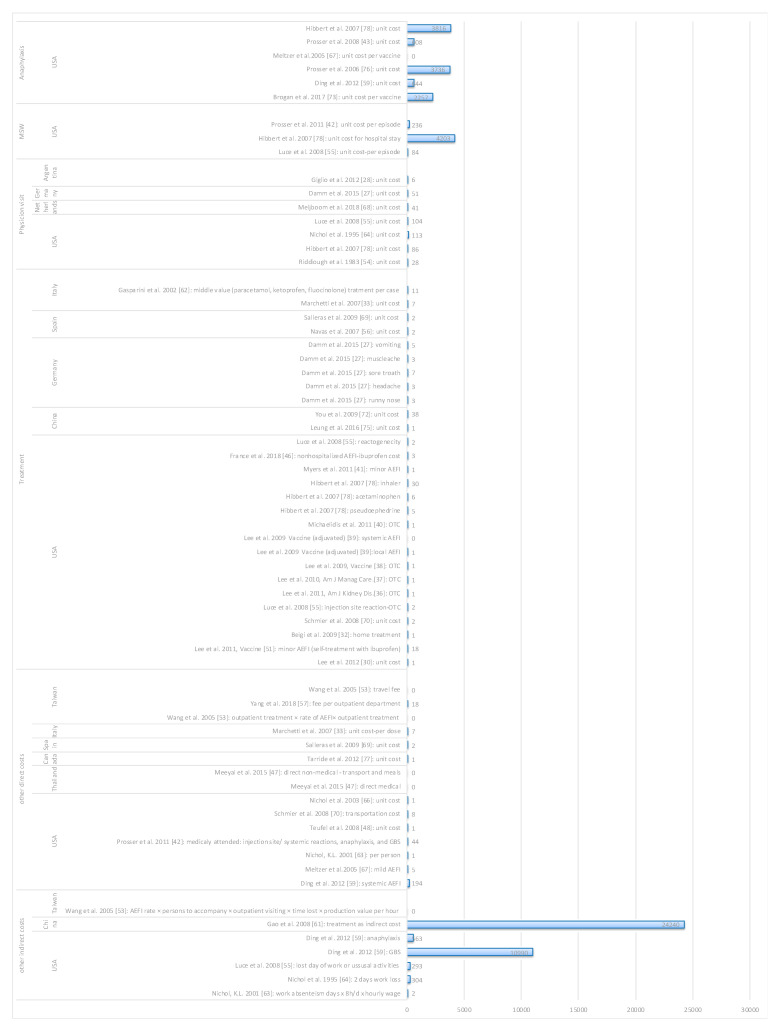
AEFI-related costs. All costs are presented in USD, and 2020 was taken as a reference price year. The cost values under 1 USD are displayed as 0 (Meltzer et al., 2005 [67]: anaphylaxis, unit cost per vaccine= 0.01; Lee et al., 2009 Vaccine (adjuvated) [39]: treatment, systemic AEFI = 0.35; Wang et al., 2005 [53]: other direct costs, travel fee = 0.02; Wang et al., 2005 [53]: other direct costs, outpatient treatment × rate of AEFI × outpatient treatment = 0.15; Meeyai et al., 2015 [47]: other direct costs, direct non-medical − transport and meals = 0.18; Meeyai et al., 2015 [47]: other direct costs, direct medical = 0.02; Wang et al., 2005 [53]: other indirect costs, AEFI rate × persons to accompany × outpatient visiting × time lost × production value per hour = 0.15).

The AEFI-related costs were dominantly included as direct medical costs in 90% (*N* = 47) of the articles [27,28,29,30,32,33,34,35,36,37,38,39,40,41,42,43,44,46,47,48,49,50,51,52,53,54,55,56,57,58,59,62,63,64,66,67,68,69,70,71,72,73,74,75,76,77,78]. In particular, costs of AEFI’s management, physician visits, hospitalizations, and medication/treatment. The indirect/broader costs of time loss seeking treatment for AEFIs, caregiver time, productivity loss/work absenteeism, traveling fees, and household costs were accounted for in 14 articles [32,42,53,55,57,58,59,61,63,64,65,66,70,71]. In Table 4, we give an example on how the costs of the most commonly included AEFI, the GBS, were presented. The costs are given per country and per article, and were given in common currency (USD as per 2020). Further overview of costs per AEFI with regards to anaphylaxis, MSW, physicians’ visits, medical treatment, and other direct and indirect costs is given in Appendix A, Figure A1.

Most of the studies reported the costs per unit, causing diversities in the presentation of cost results. For example, one study [63] calculated unit costs as follows:

- direct AEFI-related unit cost = [(number of health care provider visits for AEFI) × cost of healthcare provider visit) + (number of cases of vaccination-associated GBS)] × cost per case of GBS;
- indirect AEFI-related unit cost = number of work absenteeism days due to AEFI × 8 h/d × hourly wage.

Another study [53] used this equitation to present unit costs:

- direct AEFI-related unit cost = payment for one outpatient treatment × rate of AEFI due to vaccination × half of these need outpatient treatment;
- indirect AEFI-related unit cost = rate of AEFI × 1/2 accompanying person × payment for 1/2 outpatient treatment × time lost × productivity loss (cost per hour);

Therefore, straightforward comparisons of AEFI costs were not possible, and these results are given per individual study (see Appendix A, Figure A1 and Supplementary Materials, Table S1).

#### 3.3.2. Other AEFI-Related Issues

AEFI-related disutility/QALY losses were considered in 18 articles [29,30,32,36,37,38,39,40,41,42,44,45,46,47,55,73,74,75], given in Table 5. These studies dominantly included the disutility associated with again the GBS after vaccination with IIV or LAIV. Other AEFI-related disutilities were reported for anaphylaxis, while comparing two IVV (TIV vs. QIV) [73] or LAIV with TIV [42], and for MSW associated with LAIV [42,55]. While emphasizing the QALY loss of the previously mentioned AEFIs, we should not overlook the low occurrence, leading to low overall disutility values. Minor AEFIs inflict QALY losses of 0.99 QALYs [41,74], or local and systemic account for QALY losses in the range from 0.80 to 0.95 QALYs [36,38,39]. There were also several articles that did not specify the AEFIs, and they used QALY value of 0.95 QALYs [30,32,40] or 0.9 [45]. Obviously, such reported values comply with the ranges of local and systemic AEFI. Some studies used direct assumptions to include the QALY losses [29,41,46], or took values from literature. Leung et al. [75] and Michaelidis et al. [40] both used the study of Lee et al. [38] as a reference source, which reported assumptions as well.

Thirteen studies reported explicit specification of duration of AEFIs [30,37,38,39,40,43,45,52,55,60,63,66,75]. The duration of the local and systemic AEFIs was reported as being 1–3 days. More specifically, in two studies, a duration of 0.75 days was reported [30,60], while in another two [40,75], a duration of 2 days was included. Furthermore, four studies for local AEFIs assigned 1 day [37,38,39,52]; for systemic, 2 [39,52] or 3 days [37,38]; and another reported 3-day duration of symptoms [45]. MSW was estimated to last about 13 symptom days [55], and accounting for productivity loss for an AEFI-related outpatient visit of 4 h [37]. Anaphylaxis accounted for 2–3 days lost (productivity loss), while GBS accounted for about 40 days lost (productivity loss) [43], and 10-day work absenteeism for AEFIs given in days per 1000 were reported [63,66].

The frequencies of occurrence of AEFIs were expressed through probabilities, incidence, rates, or relative risk mentioned in 35 of the included studies [27,28,29,30,31,32,33,34,36,38,39,40,41,42,43,44,45,46,49,51,52,55,56,57,59,61,63,64,68,72,73,75,76,77,78]. The majority of them took 1% probability of occurrence of AEFIs, considering a consultation with a physician or systemic AEFIs [28,31,43,44,49,59,72,76]. It seemed like the probability for serious AEFIs from LAIV (2 × 10^−5^) was bigger than that from IIV (3 × 10^−6^), while the serious AEFIs acquiring admission to an intensive care unit was the same for both vaccines (0.33) [51]. Similarly, the reactogenicity after receiving LAIV (≈0.5) was larger than after receiving TIV (≈0.4) [55,77]. Typically reported AEFI-probabilities from LAIV were runny nose, headache, fever, sore throat, muscle ache, and vomiting [27,78]. Trial studies showed that TIV causes fever, sneezing, cough, vomiting, erythema [52], and significant difference was shown only for arm soreness, showing almost three times higher probability than in the placebo group [64].

#### 3.3.3. AEFIs’ Share of the Total Costs Discussed in the Economic Evaluation

Not all papers allowed for an estimation of the share of AEFIs in the total costs discussed in the economic evaluation. We identified only four articles [52,56,69,73] where it was possible to give a clear reflection of AEFIs’ direct medical costs in the total direct medical costs. In three of these papers, direct costs of AEFIs reflected less than 1%, and one less than 2% of total direct costs. They concern the vaccine-related AEFI management [73] and treatment of AEFIs [52,56,69], representing 0.05%, 0.34%, 0.79%, and 1.8% of the total direct costs, respectively.

### 3.4. Quality Assessment Using CHEC Extended List

**Figure A2:**
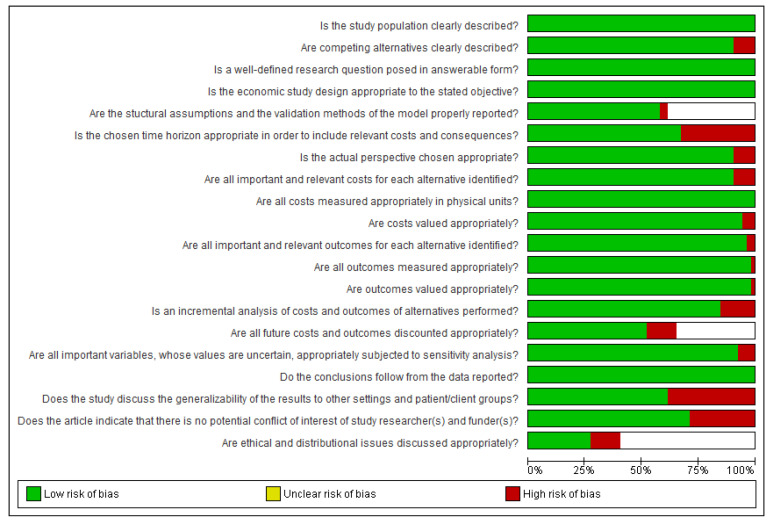
Review of authors’ judgments about quality assessment items in Consensus Health Economic Criteria (CHEC) extended list presented as percentages across all included studies.

**Figure A3:**
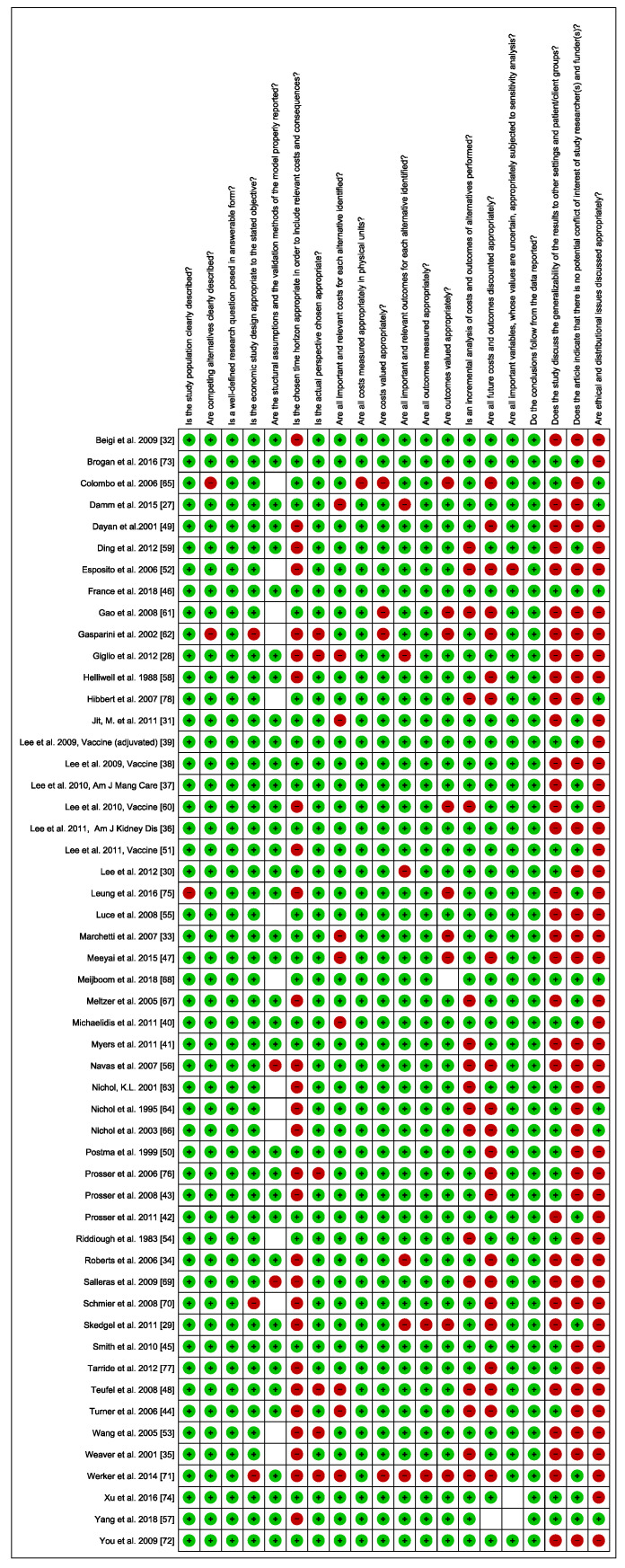
Risk of bias for each individual study, displaying the 20 questions from the Consensus Health Economic Criteria (CHEC) extended list for quality assessment of economic evaluations.

The graphical presentation of the judgments about the quality assessment items from the CHEC extended list is given in Appendix A, Figure A2. This assessment showed that the generalizability is worst reported. Moreover, the time horizon reporting reflected poor results. As the majority of the studies reported time horizon of one year or one season, we found that period not long enough to capture all the AEFI outcomes if the study was not in trial settings, or especially if it considered the societal perspective. On the other hand, all articles had well-defined research questions and appropriate study design, clearly described the study population, and properly presented the conclusions following the reported data. Overall, we found the reporting quality of the papers to be at a satisfactory level. Risk of bias for each individual study accounting the 20 questions form the CHEC extended list for quality assessment of economic evaluations is given in Appendix A, Figure A3.

## 4. Discussion

### 4.1. Main Findings

**Figure 2:**
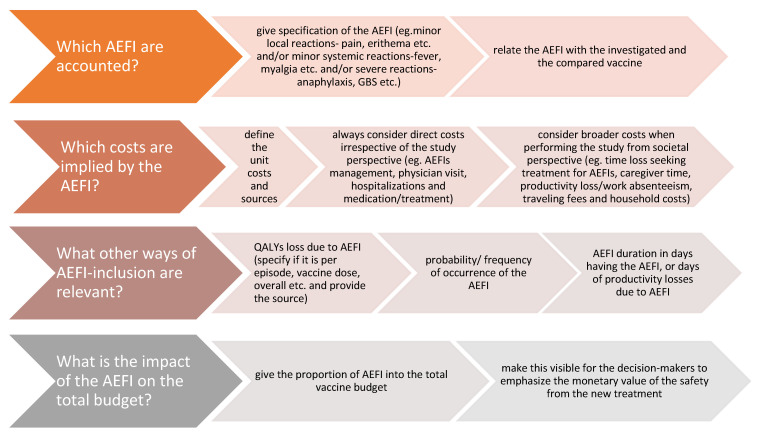
A four-step structure for inclusion of AEFI within economic evaluation on seasonal influenza vaccines. AEFI—adverse event following immunization; QALYs—quality-adjusted life years.

We conducted this study to investigate if and how the economic evaluations on seasonal influenza vaccines include the AEFIs, and further explored how are they used in the analyses considering this issue. The results showed that 16% of the studies we initially considered for full-text screening included effects of AEFI within the economic evaluation. However, among the studies that accounted for AEFI, no consistent and comprehensive reporting of AEFI was noted. In this matter, costs and utilities of rare and expensive AEFIs, such as GBS, seemed to be more interesting for inclusion in the economic evaluation on influenza vaccines than the more frequently occurring but mild AEFIs. While the reporting of AEFIs costs was preferred in a matter of direct costs, the indirect/broader costs were not presented in all studies from societal perspective. The impact of AEFI costs into total study costs was shown to be minimal and not always easy to estimate. Summarizing and analyzing the outcomes of this study, we propose a “four steps structure” (Figure 2) that can serve as an indicator for better and more comprehensive inclusion of the AEFIs while performing economic analyses on seasonal influenza vaccine.

### 4.2. Interpretation

The three main reasons for not including the effects of the adverse events (AE) within the economic evaluations are referring to inconsequential differences between the compared options, minor influence on the quality-of-life, or lack of relevant data. Such approaches potentially deprive the right of decision makers and, indirectly, the target population for the vaccine intervention to be informed about the safety parameters and possible costs related to it. As the reporting of the cost-effectiveness is performed either from the payer perspective or the societal perspective, most reported costs in both perspectives account for direct medical and non-medical cost. Despite the common acceptance of their inclusion, indirect costs remain less reported than direct costs [82] also in the societal context of our reviewed studies. Such a situation is potentially imposed by the variations in national requirements regarding the pharmacoeconomic guidelines and costs or utilities to be considered [83].

It is to be expected that the serious events require longer or more complicated treatment and account for higher costs. Similarly, the local and less serious systemic AEFI would report more modest cost, as those usually included costs of over-the-count medicines. It can also happen that some of the systemic AEFIs require hospitalizations, which escalates the AEFI-related costs. What we found paradoxically in this situation, observing our reviewed studies, was having the GBS as the most commonly reported among the reviewed studies [29,34,41,42,43,47,59,63,73,74,78], in a matter of costs, while knowing that those AEFIs are least likely to occur, 1 in 1,000,000 (vaccine associated probability) [84], or 0.8–1.9 in 100,000 person/year (population incidence) [85,86]. This AEFI was for the first time emphasized as an AEFI after receiving influenza vaccine back in the 1970s [87] and remains noteworthy until today. It might be worthwhile mentioning that all the studies that reported GBS-related costs and utilities considered the American concept, implying potential role of the population size (higher absolute number of people with AEFI) in the determination of AEFI inclusion. Furthermore, we believe that the high number of inclusions of the GBS is the result of the costly management of this severe event. However, this AEFI is not the one people will usually encounter.

The utility data on AE presented in the economic evaluations can be derivates either from direct observation on patients that had the intervention, or from the literature [88]. Some literature sources from the studies in our review accounting for AEFI-related utilities led to the conclusion that the main source of the utilities is an assumption [38,40,75]. Furthermore, specifying the derivation of utilities is important, as it might occur that studies already incorporated the AEFI impact on the quality of life if the values were derived from the intervention that already accounted for AE [88]. This is particularly important if we want to have the information on specific utility, such as the AEFI-related utility, the one of interest in our research. Seen from some previous reviews, this was not the case, as they all accounted for overall QALY gains or losses [89,90,91]. With these data in mind, it should be also noted that AEFIs causing large QALY loss have the lowest probabilities of occurrence. Safety and efficacy data, as well as probabilities of occurrence and AEFI duration studies are usually combined in a trial. A survey of current practice [88] showed that the majority of the AEFIs were derived from clinical data. Each medication prior registration is subject to pre-clinical safety and efficacy trial. Such derived data are further used while performing economic evaluations on the comparing interventions. Of the same height, study-based (trial/observational/survey) economic evaluation, included in our review [35,43,44,52,53,54,56,57,61,62,63,65,66,68,70,71,72,77,78,92], gave more explicit information on the AEFI than the model-based studies, which are more likely to account for parameter assumptions.

Estimating the share of AEFI-related costs into the total costs turned out to be a challenge. First, not all articles presented such data, and some even restricted their AEFI cost-data presentation by reporting it descriptively [31,44,45,60,71]. Second, we could only estimate the share of AEFI related cost that were part of the direct costs, impacting the total direct costs with less than 2% [52,56,69,73]. If this number is further used to make an estimate for the total budget, it will show an even smaller impact. This implies very modest costs of the included AEFIs in the economic evaluations on seasonal influenza. In this matter, Luce et al. [92] and Allsup et al. [93] intended to include the AEFIs in their cost analysis, but the results from the clinical trial showed no noteworthy outcome to be considered in the costs, and for that reason they excluded it from further analyses. On the other hand, Gatwood et al. reported that the economic impact of the costs of moderate AEFI is often reported, but showed considerable variation [94].

A brief review showed a scarce interest in exploring the effects of AEFIs within economic evaluations. Only one “older” survey study explored the incorporation of adverse effects in overall economic models, published between 2004 and 2007, and suggested clearer and explicit reporting [88]. Our review supports this statement, and it is the first to systematically address this issue in the context of economic evaluations on seasonal influenza vaccines. Previous reviews mainly focused on particular vaccine type or target population group, while we explored all types of seasonal influenza vaccines. Hence, de Boer et al. [91] and Thommes et al. [95] explored the effects of QIV, while Loperdo et al. [96] focused on TIV adjuvated vaccines, emphasizing the importance of age groups in selecting the vaccine type. Yet, no particular attention was brought on AEFI inclusion. Similarly, with no attention on AEFI, a review showed that children’s vaccination is a cost-effective intervention [97] while emphasizing that they represent an important group in influenza transmission [98,99]. In the context of reporting on children’s vaccination with seasonal influenza vaccine, our analysis showed that the economic evaluation including safety/AEFI-related parameters concerned most frequently the analysis on children. Needless to say, the recent reviews on seasonal influenza vaccine did not explored inclusion of AEFI, and therefore direct comparison to those studies was not possible. However, a review on seasonal influenza vaccines for healthcare workers reported that studies provide insufficient data to assume the effects of AEFI, emphasizing the need for their inclusion [100]. Moreover, a recent study went beyond the economic evaluation concept to investigate the public’s view on vaccination strategies, including influenza vaccine, and showed that the public weight one averted AEFI equally to tree disease infections in children [101].

### 4.3. Strengths and Limitations

To the best of our knowledge, this is the first review on economic evaluations, mapping the AEFI-related issues on seasonal influenza vaccines, with no time, language, or target population limitation. It provides comprehensive insides of the safety-related parameters and structures them to facilitate their use into future studies. Moreover, the review followed the recommended reporting guidelines for performing a systematic review, followed the published protocol in PROSPERO, and adhered to PRISMA. Additionally, our reporting is in line with the five-step approach for conducting reviews on economic evaluations of Mastrigt et al. [83]. While other reviews on economic evaluations reported ICERs and net savings per vaccine [89,90,91], we performed a unique review on economic evaluations where the end points were the AEFI-related costs and other AEFI- outcomes, for example, health-related quality of life.

That said, the review has certain limitations. First, presenting the outcomes into meta-analysis could have brought a valuable contribution in to the future development of economic evaluations of seasonal influenza vaccines. Such analysis would have allowed us to quantify and characterize the AEFI outcomes and explore their reliability and validity. Yet, this is more common practice when performing record reviews [102,103]. Moreover, the high heterogenicity of the reported parameters, resulting from the different national requirements and policies for performing economic evaluation, did not allow such a design [100]. Therefore, all results were presented individually, and the extracted cost parameters converted in a common currency and same price year [83].

Second, the tool we used to assess the risk of bias in each economic evaluation did not include items on AEFI, or safety in general. However, we used the CHEC extended list [22,23] as it is appropriate for appraisal of trial-based and model-based economic evaluations [83], which we both included in this review. Moreover, none of the existing tools for assessing the risk of bias among the economic evaluation (such as the guidelines for authors and peer reviewers of economic submissions to the British Medical Journal (BMJ) [104], Phillips checklist [105], or the International Society for Pharmacoeconomics and Outcomes Research (ISPOR) checklist [105]) address the AEFI issues, implying an update of these tools accordingly [106].

Third, while presenting the cost outcomes, it was not always easy to assign them a straightforward category. Those costs we assigned into wider category of other direct and other indirect costs. Moreover, the expression of unit costs was altered to the needs of the study itself and may lead to incorrect interpretation if comparing unit costs of different studies. Therefore, in our presentation, we referenced each cost. Apropos our secondary outcome, we were not able to make a share estimation of the AEFI costs in the total budget, but only in the total direct costs. This might have been the result of the frequent inclusion of AEFIs as direct costs, but also due to the fact that these costs are not being considered as highly influential in the final budgets [92,93]. For example, one study [53] presented costs for AEFI to direct costs with a value of 0.104, and in indirect cost 0.107; still, it was not clear if the costs of the hospitalization were concerning the AEFI, and thus we did not calculate their share in the total costs. Another study [63] gave values for direct (0.61) and indirect (1.47) costs of AEFI, but it was not clear what value to use for total costs since the cost for the vaccine and its administration was unknown, and there were two ways to assume this value, which would have brought us to different outcomes. In another study [62], calculated costs for AEFI turned to be 1.09% in the total vaccination (not specified direct and indirect costs) costs concerning nine patients receiving three different anti-inflammatory medications. To this end, we decided to solely include the four articles [52,56,69,73] to avoid further misinterpretation.

### 4.4. Research Implications

Serious AEFIs do not always occur immediately after vaccination. Follow-up within two weeks after vaccination with seasonal influenza vaccine should be sufficient to observe the common AEFI [107]. However, the serious events, such as the GBS, will not be considered within that observation time. The GBS may occur in five [87] or six [108] weeks after vaccination. Moreover, when the trial size is relatively small, we face the problem of not capturing the rare AEFI. This might be an indicator for the future performers of economic evaluations of seasonal influenza vaccine to think of the AEFI observation time as an issue that can indicate more or less costly AEFI to be considered for inclusion in the analysis, or account for real-world data on AEFI. In addition, larger-size-trials should be conducted in future in order to allow manifestation of the rare AEFI.

While the public accounts more weight to the AEFI than to the infection itself [101], within the economic evaluations, the effects of the AEFI seem to matter less. Additionally, studies often used assumptions to express the health-related quality-of-life per AEFI, showing necessity for more studies evaluating such health outcomes due to the AEFI.

Furthermore, an update of the existing guidelines for economic evaluation is needed to strengthen the requirements for inclusion of AEFI. In line with this, the tools assessing the risk of bias in the economic evaluations should also be updated to account for safety-related items. Moreover, we urge to emphasize the importance of coordination between vaccine and pharmacoeconomic guidelines to better utilize the AEFI reporting in economic evaluations. This will ensure a complete transparency and comprehensive analyses when safety is concerned.

In future, creation of online interactive platform for displaying categories of AEFI data, where researchers can input their AEFI-related data, or find AEFI data, would allow easy access and potently increase the inclusion of safety-related issues into economic-evaluations on seasonal influenza vaccines.

## 5. Conclusions

Our study showed that 16% of the initially eligible full-text articles considered AEFI-related costs, utilities, frequency, or duration in the modeling, while the rest limited the inclusion to discussion only or excluded AEFIs because of assuming equality/similarity when comparing two vaccines. Direct costs, mostly from rare AEFIs, such as GBS, appeared to be the most commonly considered in economic evaluations of seasonal influenza vaccines. Total share of its cost is minimal, but important for comprehensive preview for the decision makers as well as increased public trust in the vaccination strategies.

## Figures and Tables

**Table 1:** Detailed search strategy.

Search Databases									
	PubMed			Embase			Cochane Library		
Search Line	MeSH Terms		Title/Abstract	Emtree Terms		Title/Abstract	MeSH Terms		Title/Astract/Key words
Influenza	influenza, human	OR	influenza OR flu	influenza, human	OR	influenza OR flu	influenza, human	OR	influenza OR flu
AND									
Vaccine	vaccines OR vaccination	OR	vaccine OR vaccines OR vaccination OR flu shot	vaccines OR vaccination	OR	vaccine OR vaccines OR vaccination OR flu shot	influenza vaccines OR vaccines OR vaccination	OR	vaccine OR vaccines OR vaccination OR flu shot
AND									
Seasonal			seasonal OR epidemic OR annual OR universal			seasonal OR epidemic OR annual OR universal			seasonal OR epidemic OR annual OR universal
AND									
Economics	“costs and cost analysis” OR “quality-adjusted life years” OR “economics” [subheading]	OR	cost OR costs OR economic evaluation OR economic analysis OR qaly OR qalys ORquality-adjusted-life-year OR quality-adjusted-life-years OR hye OR healthy years equivalent OR daly OR disability-adjusted life years OR icer OR icur OR work productivity OR absenteeism	“cost”/exp OR “quality adjusted life year”/exp	OR	cost OR costs OR “economic evaluation” OR “economic analysis” OR qaly OR qalys OR “quality-adjusted-life-year” OR “quality-adjusted-life-years” OR hye OR “healthy years equivalent” OR daly OR “disability adjusted life years” OR icer OR icur OR “work productivity” OR absenteeism	[cost and cost analysis] OR [quality-adjusted life years] OR any MeSH descriptor with qualifier(s): [economics-EC]	OR	cost OR costs OR economic evaluation OR economic analysis OR qaly OR qalys OR quality-adjusted life year OR quality-adjusted life years OR hye OR healthy years equivalent OR daly OR disability-adjusted life years OR icer OR icur OR work productivity OR absenteeism

MeSH (medical subject headings); QALY (quality-adjusted life years); HYE (healthy years equivalent); DALY (disability-adjusted life years); ICER (incremental cost-effectiveness ratio); ICUR (incremental cost-utility ratio).

**Table 2:** Summary of study characteristic results from the 52 evaluated studies.

Study Characteristic	Study Characteristic in the Reviewed Article	Percentage of Studies
Type of EE	CBA	21%
	CEA	21%
	CUA	38%
	CMA	0
	combining more than one type	19%
Study perspective	societal	35%
	combining more than one perspective	29%
	healthcare provider	12%
	employer	4%
	family	2%
	medical insurance	2%
	school	2%
	patient	2%
	net monetary benefit	2%
	not specified	12%
Vaccine type	LAIV vs. no vaccination or alternative vaccine	15%
	IIV vs. no vaccination or alternative vaccine	85%
Population characteristics	general population	4%
	HIV population	2%
	haemophilysis population—adults	2%
	pregnant/pospartum woman and infant	13%
	children	35%
	adults 18–49 y standard risk	2%
	adults 50 y	2%
	adults healthy 50–64 y	2%
	adults 18–65+ y	2%
	working adults age/employees elderly 65+ y	13% 23%
Location	USA	56%
	China	6%
	Thailand	2%
	Taiwan	4%
	Gemany	2%
	Argentina	4%
	Canada	8%
	UK	4%
	Netherlands	4%
	Spain	4%
	Italy	8%
Settings	model-based	62%
	study-based (trial/observational/survey)	38%
Funding	no information regarding funding	31%
	not funded funded not specified	6% 63% 4%
	research/training fund or grant	6%
	National Institute/fund/Ministry	27%
	CDC	9%
	WHO pharmaceutical company/vaccine manufacturer	2% 15%
Type of AEFI	severe AEFI (GBS, anaphylaxis, and MSW)	33%
	minor/mild AEFI (local and systemic)	46%
	AEFI not specified	40%
AEFI inclusion	direct costs of AEFIs	90%
	indirect costs of AEFIs	27%
	disutility/QALY-loss due to AEFIs	37%
	duration of AEFIs	21%
	probability/frequency of occurrence of the AEFIs	54%

EE—economic evaluation; CBA—cost–benefit analysis; CEA—cost-effectiveness analysis; CUA—cost–utility analysis; CMA—cost-minimization analysis; LAIV—live attenuated influenza vaccine; IIV—inactivated influenza vaccine; WHO—World Health Organization; CDC—Center for Disease, Control and Prevention; AEFI—adverse event following immunization; QALYs—quality-adjusted life years; GBS—Guillain–Barré syndrome; MSW—medically significant wheezing. The lighter points are subgroup of funded studies.

**Table 3:** Vaccine alternatives compared and AEFI outcomes reported within the 52 studies.

Vaccine Type	Studio Vaccine	Comparator	AEFI-Related Costs	Other Forms of AEFI Inclusion in the EE	Study Identifica-tion
**inactivated influenza vaccines**	QIV	TIV	- vaccine-related AEFI (anaphylaxis and GBS)	- QALY loss (anaphylaxis and GBS) - frequency (anaphylaxis and GBS)	Brogan et al., 2017 [73]
	seasonal vaccination	no vaccination	- vaccine -related AEFI (GBS) - medications	- QALY loss (minor AEFI and GBS)	Xu et al., 2016 [74]
	IIV administrated intradermally	IIV administrated intramuscularly	- treatment (injection site reaction, headache, and myalgia)	- probability of AEFI - relative risk - QALY loss - duration (days)	Leung et al., 2016 [75]
	TIV	trivalent LAIV	- direct medical and non-medical cost (GBS) - indirect or time cost due to seeking treatment	- DALY loss per vaccine dose	Meeyai et al., 2015 [47]
	TIV injectable	LAIVnasal spray	- direct unit cost - treatment of LAIV-associated AEFI (GP consultation, runny nose, headache, fever, sore throat, muscle aches, vomiting)	- probability of LAIV-related AEFI (based on the absolute differences between LAIV and placebo observed in clinical trials within 10 days after the first dose)	Damm et al., 2015 [27]
	seasonal vaccine	no vaccination	- costs of AEFI as part of public investment for pediatric influenza vaccination (GP consultation)	- probability of AEFI	Giglio et al., 2012 [28]
	TIV injectable	no vaccination	- direct costs (systemic reaction, anaphylaxis, GBS) - indirect nonmedical costs (anaphylaxis days lost, GBS days lost)	- probabilities (local reaction, systemic reaction, anaphylaxis, GBS)	Ding et al., 2012 [59]
	universal vaccine	no vaccination	- annual cost (GBS)	- relative utility weight (GBS) - relative risk (GBS)	Skedgel et al., 2011 [29]
	universalvaccine	standard annualvaccine	- treatment of vaccine AEFI	- duration (time after having vaccine AEFI) - QALY loss - probabilities	Lee et al., 2012 [30]
	TIV	LAIV	- minor AEFI (local inflammation or minor systematic flu-like symptoms, requiring self-treatment with ibuprofen), - hospitalization for major AEFI (GBS), - ICU (for major AEFI) for commercial insured and uninsured	- probabilities (major AEFI, major AEFI requiring ICU, and minor AEFI)	Lee et al., 2011, *Vaccine*, [51]
	seasonal vaccine	no vaccination	- no specific cost data related to AEFI were presented	- probability of mild AEFI in adults - occurrence of systemic AEFI (equivalent to having influenza for a single day)	Jit et al., 2011 [31]
	seasonal vaccine	no vaccination	- home treatment	- QALY loss - probability per dose	Beigi et al., 2009 [32]
	subunit vaccine	no vaccination	- unit costs per AEFI	NA	Salleras et al., 2009 [69]
	seasonal vaccine	no vaccination	- medical expenses for treatment of AEFI	- occurrence of AEFI	Gao et al., 2008 [61]
	virosomal subunit vaccine	no vaccination	- unit costs per AEFI	- occurrence (local and mild)	Navas et al., 2007 [56]
	virosomal adjuvanted vaccine	current immunization (TIV and LAIV)	- healthcare cost (per dose for fever)	- occurrence (fever) - relative rate (fever)	Marchetti et al., 2007 [33]
	TIV	no vaccination	- costs for GBS - medications for AEFI	- probability (GBS and medical visit)	Roberts et al., 2006 [34]
	TIV (2 doses), virosome-formulated subunit vaccine	no vaccination	- direct costs requiring pharmacological treatment	- occurrence (*systemic events*: fever, sneezing, cough vomiting; *local events*: erythema/tenderness) - durations	Esposito et al., 2006 [52]
	universal vaccine	no vaccination	- direct costs (treatment) - -indirect costs (work productivity and traveling fee)	NA	Wang et al., 2005 [53]
	annual vaccine	no vaccination	- total cost per vaccine (for mild AEFIs, GBS, and anaphylaxis)	NA	Meltzer et al., 2005 [67]
	influenza vaccine	no vaccination	- pharmacological treatment (administration of paracetamol, ketoprofen, fluocinolone acetonide)	- occurrences of AEFI	Gasparini et al., 2002, [62]
	influenza vaccine	no vaccination	- direct costs (medical care costs including healthcare provider visit, tests, and medications) and direct costs per episode (GBS) - indirect costs (work absenteeism)	- duration: work absenteeism (days) - healthcare provider visits (number of visits) - GBS occurrence	Nichol, K.L., 2001 [63]
	influenza vaccine	no vaccination (placebo vaccination)	- direct costs (medical care, office visit) - indirect costs (work loss)	- occurrence rate (fever, tiredness, feeling “under the weather”, muscle aches, headaches, arm soreness)	Nichol et al., 1995 [64]
	annual influenza vaccine	no vaccination	- treatment (any AEFI and GBS)	- utilities lost (minor: fever, malaise, myalgia; immediate: respiratory difficulties, skin eruptions; systemic reactions: assumed that a reaction would entail one day of non-bed disability and GBS)	Riddiough et al., 1983 [54]
	annual vaccine	no vaccination	- per immunization	NA	Helliwell et al., 1988 [58]
	influenza vaccine	no vaccination	- outpatient visit	- incidence of non-bad disability day - incidence of outpatient visit	Weaver et al., 2001 [35]
	adjuvanted vaccine	non adjuvanted vaccine	- treatment (OTC medications)	- utilities (QALYs) mean - probabilities for AEFI from vaccine without adjuvant and with adjuvant	Lee et al., 2011, *Am J Kidney Dis.* [36]
	influenza vaccine	adjuvated influenza vaccine	- treatment of vaccine side effects (local and systemic AEFI)	- durations (local and systematic AEFI) - utilities (QALYs for local- and systematic AEFI) - probabilities (local and systematic AEFI)	Lee et al., 2009, *Vaccine* (adjuvated) [39]
	influenza vaccine	no vaccination	- treatment	- probability (per annual event) - duration (days) - utilities (QALY)	Michaelidis et al., 2011 [40]
	TIV	no vaccination	- minor AEFI - GBS	- probabilities (minor AEFI and GBS) - utilities (minor AEFI and GBS)	Myers et al., 2011 [41]
	(1) traditional physician office (2) mass vaccination (3) pharmacy setting	no vaccination	- physician visit for systemic reaction - anaphylaxis, medical costs - GBS, medical costs	- probabilities (local reaction, systemic reaction, anaphylaxis, GBS) - productivity loss (days due to GBS)	Prosser et al., 2008 [43]
	influenza vaccine	no vaccination	- cost per AEFI per vaccine	NA	Teufel et al., 2008 [48]
	TIV non-adjuvated seasonal influenza vaccine	the vaccine is compared in different periods	- cost for a single dose	NA	Werker et al., 2014 [71]
	(1) IV (annually) + PCV (on 5 y)(2) IV annually (3) no vaccination	comparing the outcomes of the three strategies	- treatment costs	- incidence of influenza vaccine AEFI	You et al., 2009 [72]
	seasonal vaccine	no vaccination	- indirect costs (working days lost)	NA	Colombo et al., 2006 [65]
	influenza vaccination	no vaccination (placebo)	- cost per QALY saved for AEFI	- probability of AEFI - QALD loss - frequency of systemic AEFI	Turner et al., 2006 [44]
	influenza vaccine	no vaccination	- treatment	- probability of AEFI	Dayan et al., 2001 [49]
	influenza vaccine	no vaccination	- GP visit (mild AEFI)	NA	Postma et al., 1999 [50]
	pneumococcal polysaccharide vaccine and influenza vaccination strategies	current CDC recommendations (influenza vaccination for all, PPV when comorbid conditions are present)	NA	- duration of symptoms (days) - probability of AEFI - utility weights	Smith et al., 2010 [45]
	influenza vaccine	no vaccination	- treatment (ibuprofen)	- probability of AEFI - disutility (QALY lost)	France et al., 2018 [46]
	influenza vaccine	no vaccination	- direct costs (per outpatient department) - indirect costs (productivity loss hours and traveling fee)	- probability of AEFI	Yang et al., 2018 [57]
	influenza vaccine	no vaccination	- GP visits	- occurrence rate	Meijboom et al., 2018 [68]
	influenza vaccine	(1) vaccination timing model- intake of vaccine at different months for estimating the timing (2) comparing monthly vaccination with no vaccination of 65+ population	- treatment of vaccine side effects	- probability (clinical outcomes) - duration (days of ibuprofen treatment for local or systemic AEFI) - utility (QALYs lost)	Lee et al., 2009, *Vaccine* [38]
	TIV LAIV	(1) children’s vaccination timing model and (2) children’s monthly influenza vaccination decision model	- treatment (ibuprofen)	- probability of experiencing AEFI - duration days of ibuprofen treatment for local or systemic AEFI) -productivity losses (hours) - utility (QALYs lost)	Lee et al., 2010, *Am J Manag Care.* [37]
**live attenuated influenza vaccine**	LAIV	TIV	- no cost data related to AEFI	- duration (days)	Lee et al., 2010, *Vaccine* [60]
	LAIV intranasal	no vaccination	- costs associated with immunization (unit cost) - costs per household during peak week and projected influenza season -per intervention - transportation (unit cost)	NA	Schmier et al., 2008 [70]
	LAIV	IIV	- physician visit (injection site reaction, anaphylaxis, GBS)	- probabilities (medically-attended AEFI for injection site, systematic reaction, anaphylaxis, and GBS)	Prosser et al., 2006 [76]
	LAIV	TIV	- per episode (MSW, reactogenicity, injection-site reaction) OTC medication	- clinical probabilities, % (MSW reactogenicity event, injection site event, emergency room visits due to MSW - health state utilities (QALY per MSW) - duration (MSW average number of symptoms days)	Luce et al., 2008 [55]
	LAIV	TIV and no vaccination	- wheezing episode (physician or emergency department visit and prescription medications, bronchodilator) - medically attended (injection site reactions, systemic reactions, anaphylaxis, and GBS)	- probability (wheezing, LAIV only and hospitalization for child (2–4 years) with wheezing - disutilities (wheezing episode, anaphylaxis, and GBS)	Prosser et al., 2011 [42]
	LAIV	TIV	- physician office visit	- clinical probabilities (MSW, injection site events, reactogenicity)	Tarride et al., 2012 [77]
	trivalent, intranasal, (LAIV)	no vaccination (placebo)	- direct cost for AEFI - indirect cost, lost time due to AEFI	- duration (days of work lost)	Nichol et al., 2003 [66]
	LAIV trivalent (nasal spray)	no vaccination (placebo)	- physician visit - hospital stays for wheezing episode - anaphylaxis treatment - GBS - treatments costs for pseudoephedrine, acetaminophen - albuterol inhaler	- vaccine reaction rates (additional cases of runny nose/nasal discharge, additional cases of fever ≥ 37.5 °C and ≥ 38.6 °C, additional cases of wheezing after each dose, anaphylaxis, and GBS)	Hibbert et al., 2007 [78]

AEFI—adverse event following immunization; EE—economic evaluation; IIV—inactivated influenza vaccine; LAIV—live attenuated influenza vaccine; TIV—trivalent influenza vaccine; QIV—quadrivalent influenza vaccine; PCV—pneumococcal conjugate vaccine; universal vaccine—vaccine targeting pervasive portion of the influenza virus, so it can potentially be used for multiple years; QALYs—quality-adjusted life years; QALD = quality-adjusted life days (1 QALY = 365 QALDs); DALY—disability-adjusted life years; GBS—Guillain–Barré syndrome; MSW—medically significant wheezing; GP—general practitioner; ICU—intensive care unit; OTC—over-the-counter; CDC—Center for Disease, Control and Prevention.

**Table 4:** Guillain–Barré syndrome—related costs.

Study Identification	Location	The Way GBS Cost Was Included	Costs
Brogan et al., 2017 [73]	USA	unit cost per vaccine	69,222
Xu et al., 2016 [74]	USA	unit cost per event	51,814
Ding et al., 2012 [59]	USA	unit cost	93,747
Lee et al., 2011, *Vaccine* [51]	USA	unit cost—hospitalization (insured)	1866
Lee et al., 2011, *Vaccine* [51]	USA	unit cost—hospitalization (non-insured)	6298
Lee et al., 2011, *Vaccine* [51]	USA	unit cost—ICU (insured)	3086
Lee et al., 2011, *Vaccine* [51]	USA	unit cost—ICU (non-insured)	12,695
Prosser et al., 2006 [76]	USA	unit cost	32,322
Roberts et al., 2006 [34]	USA	unit cost for each treatment—probability weighted average	135,743
Meltzer et al., 2005 [67]	USA	unit cost per vaccine	0.352
Nichol, K.L., 2001 [63]	USA	cost per episode per vaccine	17,767
Riddiough et al., 1983 [54]	USA	net cost for medcare program	0.027
Myers et al., 2011 [41]	USA	unit cost	48,999
Prosser et al., 2008 [43]	USA	unit cost	84,709
Hibbert et al., 2007 [78]	USA	unit cost	33,033
Skedgel et al., 2011 [29]	Canada	annual cost	130,798

All costs are presented in USD, and 2020 was taken as the reference price year. GBS—Guillain–Barré syndrome; ICU—intensive care unit.

**Table 5:** AEFI-related disutility among economic evaluations on seasonal influenza vaccine.

AEFI 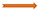		GBS	Anaphylaxis	MSW	Other
**Study Identification**	Brogan et al., 2017 [73]	QALYs loss per AE = 0.141	QALYs lossper AE = 0.020	/	/
	Xu et al., 2016 [74]	health utility index = 0.5	/	/	health utility index for minor AEFI = 0.99
	Leung et al., 2016 [75]	/	/	/	utility loss = 0.05
	Meeyai et al., 2015 [47]	DALYs loss per vaccine dose = 3 × 10^−8^	/	/	/
	Skedgel et al., 2011 [29]	relative utility weight = 0.25	/	/	/
	Lee et al., 2012 [30]	/	/	/	utilities (QALYs) = 0.95
	Beigi et al., 2009 [32]	/	/	/	utilities (QALYs) = 0.95
	Lee et al., 2011, *Am J Kidney Dis.* [36]	/	/	/	utilities (QALYs) = 0.95
	Lee et al., 2009, *Vaccine* (adjuvated) [39]	/	/	/	utilities (QALYs) = 0.80 for systematic AE utilities (QALYs) = 0.95 local vaccine AE
	Michaelidis et al., 2011 [40]	/	/	/	utility per day (QALY) = 0.95
	Myers et al., 2011 [41]	utility = 0.5	/	/	utility = 0.99 for minor AE
	Turner et al., 2006 [44]	/	/	/	QALD loss = 0.55
	Smith et al., 2010 [45]	/	/	/	utility weights = 0.9
	France et al., 2018 [46]	/	/	/	QALY loss = 0.00274
	Lee et al., 2009, *Vaccine* [38]	/	/	/	utility (QALYs) = 0.95
	Lee et al., 2010, *Am J Manag Care* [37]	/	/	/	utility (QALYs) = 0.95
	Luce et al., 2008 [55]	/	/	health state utility = 0.085	/
	Prosser et al., 2011 [42]	quality adjustments (disutility associated with an event) = 0.141	quality adjustments (disutility associated with an event) = 0.02	quality adjustments (disutility associated with an event) = 0.0018	/

AEFI—adverse event following immunization; AE—adverse event; QALYs—quality-adjusted life years; QALD = quality-adjusted life days (1 QALY = 365 QALDs); DALY—disability-adjusted life years; GBS—Guillain–Barré syndrome; MSW—medically significant wheezing; QALY values of one were ascribed for perfect health condition, and values of zero for death. Expressing disutility/QALY loss reflects the proportion of health reduced from 1 (a perfect state of health).

## Supplementary Materials

The following are available online at https://www.mdpi.com/2076-393X/9/2/111/s1, Table S1: Data extraction.

## Appendix A

This appendix provides tables and figures that complement the text presented in the manuscript.

**Table A1:** Study Selection Form.

Study Selection Form				
Reviewer: Date: Author: Title: Year: Record Number:				
	**Yes**	**No**	**Unclear**	**Not Applicable**
1. Is the article a full economic evaluation study (designs to be considered: CMA, CEA, CBA, or CUA)?	□	□	□	□
2. Is the intervention a vaccination?	□	□	□	□
3. Is the vaccine used for seasonal influenza?	□	□	□	□
4. Are the outcome measures economic parameters?	□	□	□	□
5. Does this EE discuses AEFI? *				
* This parameter will be considered only in full text selection/eligibility. If yes or unclear, final eligibility will be performed on the basis of full-text. CEA = cost effectiveness analyses; CBA = cost–benefit analysis; CUA = cost–utility analysis; EE = economic evaluation; AEFI = adverse events following influenza				

**Table A2:** Kappa statistics inputs.

Reviewer 1		Reviewer 2	
Yes	No	Yes	No
30	167	27	170
a	b	c	d

Kappa Statistics—Calculations: *N* = 197, Agreement = (a + d)/n = 1.015, Reliability Cohens kappa = (P_0_ − P_e_)/ 1 − P_e,_ P_0_ = (a + d)/n = 1.015, P_e_ = {(a + c)(a + d)/n + (b + d)(c + d)/n} / n = 2.004, Reliability Cohen’s kappa = (P_0_ − P_e_)/ 1 − P_e_ = 0.98.
